# Compulsivity and Treatment Resistant Schizophrenia

**DOI:** 10.1192/j.eurpsy.2025.144

**Published:** 2025-08-26

**Authors:** E. Fernández-Egea

**Affiliations:** University of Cambridge, Cambridge, United Kingdom

## Abstract

**Abstract:**

Obsessive-compulsive symptoms (OCS) are a prevalent and under-recognized complication of clozapine treatment in schizophrenia, with significant implications for clinical practice and patient outcomes. This synthesis of four longitudinal studies explores the interplay between psychosis, clozapine dose, and OCS, emphasizing their phenomenology, prevalence, and impact.

OCS, particularly checking compulsions, affect up to 47% of clozapine-treated patients, with risk factors including psychosis severity, duration of clozapine therapy, and antiserotonergic properties of the drug. A two-phase model of OCS development is proposed: during active psychosis, compulsions emerge as goal-directed responses to hypervigilance, while post-remission, they persist as maladaptive habits mediated by clozapine’s serotonergic effects. While clozapine improves psychotic symptoms, its dose and plasma levels are positively correlated with OCS persistence after psychosis remission, suggesting a dose-dependent effect.

Notably, OCS significantly reduce subjective well-being independently of depressive and psychotic symptoms but do not impair general functioning, highlighting the ego-syntonic nature of compulsions. Screening for and addressing OCS in clinical practice is critical for optimizing therapeutic outcomes.

This integrated perspective advances our understanding of the nuanced relationship between schizophrenia, clozapine treatment, and OCS, with implications for personalized treatment strategies.

**Disclosure of Interest:**

E. Fernández-Egea Grant / Research support from: Dr Fernandez-Egea is supported by the 2022 MRC/NIHR CARP award (MR/W029987/1) and this research was supported by the NIHR Cambridge Biomedical Research Centre (BRC-1215-20014). The views expressed are those of the author(s) and not necessarily of the NIHR or the Department of Health and Social Care. , Consultant of: EFE has received consultancy honoraria from Boehringer-Ingelheim (2022), Atheneum (2022) and Rovi (2022-24), speaker fees by Adamed (2022-24), Otsuka (2023) and Viatris (2024) and training and research material from Merz (2020) and editorial honoraria from Spanish Society of Psychiatry and Mental Health (2023-).

